# Dentists’ Appointment Book

**Published:** 1889-01

**Authors:** 


### Dentists' Appointment Book.

S. I. Pearson & Co., of Kansas City, Mo., have issued a very neat and convenient form of appointment book for dentists. It is very neat, compact, complete, and so small that it can be carried easily in the vest pocket, and it is so arranged as to receive correctly and fully the appointments with a mark for the alterations. We know of nothing in so small a compass as this little work, and it will be a matter of convenience to every dentist to have it either upon his operating case or in his pocket. It is very neat, convenient, and cheap. Price 50 cents.

